# Should Nurses Smoke?

**Published:** 1921-03-19

**Authors:** Gladys M. E. Leigh


*To the Editor of* The Hospital.

### Should Nurses Smoke?

*To the Editor of* The Hospital.

Sir,—The discussion "Should Nurses Smoke?" is merely a survival of the tradition that nurses are a peculiar people. The public is not intrigued as to whether women doctors, women lawyers, women dentists shall, when not engaged in attending to their patients or clients, enjoy the privileges of ordinary citizens; it merely singles out nurses as being deficient in discretion and mentally incompetent to differentiate between what is expected of them when "on duty" and what is permissible during hours of leisure. No trained certificated nurse would dream of attending to a patient with a cigarette between her lips; it would be contrary to the ethical standards of her training-school. The present discussion is neither profitable nor amusing, and the most satisfactory answer is undoubtedly "that during her hours of leisure a nurse is entitled to exercise the privileges of ordinary citizenship, and smoke or not as she feels inclined."—Yours faithfully,

Gladys M. E. Leigh.

